# Advancing Remission in Severe Asthma With Benralizumab: Latest Findings, Current Perspectives and Future Direction

**DOI:** 10.1111/cea.70083

**Published:** 2025-05-30

**Authors:** Renaud Louis, Marek Lommatzsch, David J. Jackson, Andrew Menzies‐Gow, Anat Shavit, David Cohen, Flavia C. L. Hoyte, Stephanie Korn

**Affiliations:** ^1^ University of Liege, GIGA I3 Research Group and Centre Hospitalier Universitaire of Liège, Department of Pneumology CHU Liege, Belgium Liège Belgium; ^2^ Department of Pneumology University of Rostock Rostock Germany; ^3^ Guy's Severe Asthma Centre Guy's & St Thomas NHS Trust London UK; ^4^ School of Immunology & Microbial Sciences King's College London London UK; ^5^ BioPharmaceutials Medical AstraZeneca Cambridge UK; ^6^ Late‐Stage Respiratory & Immunology AstraZeneca Gaithersburg Maryland USA; ^7^ Division of Allergy and Clinical Immunology, Department of Medicine National Jewish Health Denver Colorado USA; ^8^ Thoraxklinik Heidelberg and IKF Pneumologie Mainz Heidelberg Germany

**Keywords:** asthma, biologics, exacerbation, inflammation, remission, treat‐to‐target

## Abstract

The introduction of biologics, such as benralizumab (an anti‐IL‐5 receptor α humanised monoclonal antibody), has made remission a feasible goal for patients with severe eosinophilic asthma (SEA). However, there are remaining research gaps and no clear consensus on the definition of remission. We consolidated *post hoc* remission data from clinical trials and real‐world studies of benralizumab in patients with SEA to gather insights on: testing different definitions; predictors of remission; the effect of comorbidities on achieving remission; remission and background medication reduction; long‐term remission patterns with benralizumab; and remission in a real‐life setting. In the SIROCCO and CALIMA Phase 3 randomised studies, patients with remission had higher baseline median blood eosinophil counts, were more likely to have a FEV_1_ of ≥ 65% predicted, had fewer exacerbations within 12 months and had lower mean ACQ‐6 scores. Compared with the overall population, patients with a history of nasal polyps were also more likely to achieve remission with benralizumab. Analyses of the BORA and MELTEMI extension studies showed that in the longer term, once remission is achieved with benralizumab, patients are likely to remain in remission with continued treatment. In the open‐label, single‐arm ANDHI‐In Practice and PONENTE studies, patients achieving remission had a shorter median time since asthma diagnosis, higher median age at asthma onset and lower median ACQ‐6 scores. The SHAMAL study and the Phase 3b ANDHI‐In Practice substudy demonstrate that remission is maintained with benralizumab even when patients reduce their background medication. Finally, the XALOC‐1 real‐world study highlights how patients with lower BMI are more likely to achieve remission with benralizumab. These findings demonstrate that achieving remission in patients with SEA is feasible with benralizumab and, in turn, inform future directions for research and treatment that includes a promising shift towards a new era of treat‐to‐target. This manuscript was supported by AstraZeneca, the manufacturer of benralizumab.

**Summary**

- Eosinophil‐targeting therapies underpin the ability to achieve remission in many patients with severe eosinophilic asthma.
- Achieving remission within 12 months leads to long‐term benralizumab benefits; some patients require longer treatment.
- Data from clinical trials/real‐world evidence with benralizumab can inform remission definitions and guide future research.

## 1 Introduction

Asthma affects over 300 million people worldwide, with approximately 5%–10% experiencing severe disease [1, 2]. Patients with severe asthma often experience poor symptom control, impaired lung function, reliance on oral corticosteroids (OCS) and an increased risk of asthma exacerbations [3, 4].

Severe eosinophilic asthma (SEA) accounts for > 80% of patients with severe asthma [5], although it is of note that current cut‐offs to distinguish eosinophilic and non‐eosinophilic asthma are arbitrary and fall within the normal range of blood eosinophils [6, 7]. Interleukin 5 (IL‐5) is a crucial cytokine involved in eosinophil development, activation and survival [5, 8]. The introduction of biologic therapies, such as benralizumab, an anti‐IL‐5 receptor α humanised monoclonal antibody that reduces eosinophils through antibody‐dependent and cell‐mediated cytotoxicity [9], has led to remission in patients with SEA becoming a feasible goal [10, 11, 12]. This mirrors similar changes in practice concerning the management of other chronic diseases, such as rheumatoid arthritis and ulcerative colitis, where achieving remission has become a primary goal of treatment [13, 14, 15].

Although the need for therapies that can provide sustained asthma remission is widely agreed, the definition is still evolving and lacks a universally accepted standard [11, 16]. The continuous evolution of remission as a concept has been driven by research and clinical insights over the last 25 years [17, 18], and there have been varied definitions in recent studies and guidelines. In 2020, Menzies‐Gow et al. used a modified Delphi method to establish expert consensus on the key components for defining remission in asthma [11]. This framework defined remission as a period lasting at least 12 months where there are no significant symptoms (as verified with a validated instrument), lung function is optimised/stabilised, both patient and healthcare provider agree on the remission status, there is no use of OCS, and no asthma exacerbations [11]. Complete remission was defined as the addition of resolved asthma‐related inflammation and, if applicable, negative airway hyperresponsiveness. The Practical Guidelines for Asthma Management (PGAM) for general practitioners, published by the Japan Asthma Society, and two papers published by the Severe Asthma Network Italy (SANI) Delphi consensus also proposed definitions for remission in asthma [19]. In June 2023, the PGAM defined remission as an Asthma Control Test (ACT) score of ≥ 23 points, an absence of exacerbations and no use of systemic steroids. In August 2023, the SANI Delphi consensus defined complete remission as no systemic steroids for the treatment of asthma, an ACT score of ≥ 20, no exacerbations and stable lung function. Furthermore, a workgroup of asthma experts, including members from US allergy, pulmonary and paediatric societies, recently published a consensus statement defining asthma remission, proposing a more stringent six‐component definition [20]. Despite variability, all remission definitions generally agree on the following three criteria: good symptom control, no exacerbations and no OCS use [21].

With the growing support of remission as an achievable treatment goal for patients with SEA, national societies have adopted this concept in their asthma guidelines. In March 2023, new asthma guidelines published by the German Respiratory Society (DGP) were the first to incorporate remission as a treatment goal [22]. These guidelines include specific criteria for asthma remission, requiring no asthma symptoms and exacerbations for at least 12 months, stable lung function and no systemic steroids for the treatment of asthma [22]. In May 2023, the Spanish Guidelines for Asthma Management (GEMA) defined remission as the absence of symptoms and exacerbations for at least 12 months, achieved without the need for systemic steroids, alongside the optimisation and stabilisation of pulmonary function [23]. The updated 2024 Global Initiative for Asthma (GINA) report also now includes mention of remission as a potential goal in asthma management [24]. Consensus on these remission definitions is important to healthcare providers and patients for several reasons, especially when considering the transition of the remission concept from clinical trials to real‐world clinical practice [16]. To reduce asthma burden and improve outcomes, asthma guidelines and clinical research recommend joint decision‐making and goal setting between patients and healthcare providers, which requires a shared understanding of asthma control, particularly given the variations in remission definitions [25].

In this article, we aim to bring together benralizumab remission data from *post hoc* analyses of various clinical trials and real‐world evidence (RWE) studies conducted in patients with SEA, in order to address the following key points: testing different definitions; predictors of remission; effect of comorbidities on achieving remission; remission and background medication reduction; which patterns of remission can be seen during long‐term treatment with benralizumab; and real‐world analysis of remission.

## 2 Methods

*Post hoc* analyses were conducted on five interventional studies, including randomised controlled trials and long‐term extension studies, plus two open‐label, single‐arm trials (Table S1) remission findings from two RWE studies were also included (XALOC‐1 and ZEPHYR‐4) (Table S2) The trial designs, eligibility criteria and primary findings of these studies have been detailed elsewhere and are briefly summarised below.

### 2.1 Interventional Studies

#### 2.1.1 Randomised Controlled Trials and Long‐Term Extension Studies

SHAMAL, the first study to prospectively investigate remission in SEA, was designed to investigate whether patients controlled on benralizumab can safely reduce their inhaled corticosteroid (ICS)/formoterol dosage (reduction arm) or maintain their dose (reference arm) without compromising asthma control and remaining exacerbation free. Patients receiving OCS were excluded. In SHAMAL, the proportion of patients meeting each composite criterion of remission (no exacerbations, < 10% deterioration in FEV_1_ and ACQ‐5 score < 1.5 or ≤ 0.75) was assessed at Weeks 32 and 48. Additionally, the number and proportion of patients meeting zero, one, two and all three remission components were reported. More than half of the patients who reduced their background medications achieved remission by Week 32 in both the reduction and reference arms, and this was maintained up to Week 48 [26]. A *post hoc* analysis was conducted to assess remission at Week 32 by ICS/formoterol dose. Patients who met all composite criteria were considered to be in remission.

In the Phase 3 SIROCCO and CALIMA studies, the efficacy and safety of benralizumab were assessed in patients with SEA [27, 28]. A *post hoc* analysis of SIROCCO and CALIMA evaluated baseline characteristics in patients who did or did not achieve remission after 12 months. Patients were assessed according to three‐component and four‐component remission, respectively. Patients on maintenance OCS (mOCS) at baseline were excluded from the analysis since, in these studies, mOCS was required to remain stable.

BORA, an extension of the SIROCCO, CALIMA and ZONDA studies, assessed the long‐term efficacy and safety of benralizumab in patients with uncontrolled SEA [29]. A *post hoc* analysis evaluated the percentage and baseline characteristics of patients achieving durable remission after receiving benralizumab for 12–24 months. Patients had to meet the following criteria to be included in this analysis: no mOCS use at baseline in the SIROCCO and CALIMA studies; completion of the SIROCCO or CALIMA studies; and transition into the BORA study continuing treatment for at least 12 additional months. Remission was evaluated at the end of the SIROCCO or CALIMA studies and after 1 year in the BORA study. Each group was assessed using two composite definitions (comprising three [CR‐3] or four [CR‐4] components) of asthma remission.

MELTEMI continued to follow patients with uncontrolled SEA who completed the BORA extension [30]. A *post hoc* analysis of MELTEMI looked at long‐term components of remission, based on remission status in BORA (MELTEMI did not capture 6‐item Asthma Control Questionnaire [ACQ‐6] scores or forced expiratory volume in 1 s (FEV_1_), such that composite remission could not be analysed); therefore, components of remission, that is, no exacerbations and no mOCS use, were analysed. Outcomes in MELTEMI were based on patients' remission status assessed at Month 6 of BORA, following 18 months of exposure to benralizumab; this was because patients transitioned into MELTEMI from BORA between Weeks 16 and 40, so there were no 12‐month data available for these patients.

#### 2.1.2 Open‐Label, Single‐Arm Trials

The AIP and PONENTE studies evaluated the potential for patients treated with benralizumab to reduce OCS use and/or background asthma medications (AIP) and OCS medication (PONENTE) [31, 32]. AIP was an extension of the Phase 3b ANDHI study, during which patients who achieved asthma control with benralizumab underwent tapering of OCS and other asthma therapies [31]. PONENTE assessed rapid OCS tapering in patients with SEA treated with benralizumab [32].

A *post hoc* analysis of AIP and PONENTE evaluated baseline characteristics and remission rates among patients who were OCS‐dependent (daily OCS dose ≥ 5 mg for ≥ 3 months). Patients were considered to be in remission if they met all three remission criteria.

Furthermore, a *post hoc* analysis of AIP evaluated remission rates after 6 months and 18 months of treatment with benralizumab (at the end of ANDHI and AIP, respectively) and the proportion of patients who reduced background medications after 18 months. Background medications were reduced in patients if they had an ACQ‐6 score of < 1.5 at the time points, along with no exacerbations and no clinically meaningful deterioration in asthma symptoms since their last visit. Patients who were OCS‐dependent or had missing data were excluded from this analysis. Patients were considered to be in remission if they met all three criteria of the composite definition.

### 2.2 Real‐World Evidence Studies

XALOC‐1 was a retrospective integrated analysis of five national studies assessing benralizumab effectiveness in a real‐world setting [33]. The analysis aimed to describe remission in patients with SEA, with or without prior biologic experience, and by baseline characteristics, over 1 year of benralizumab treatment, evaluating the percentage of patients meeting the individual components as well as composite definitions of remission. Another analysis of XALOC‐1 aimed to assess the impact of body mass index (BMI) on remission [34].

Additionally, in an extension study of the retrospective UK Benralizumab Patient Access Programme (BPAP) study, which formed part of the XALOC programme, long‐term real‐world data highlighted the impact of comorbidities on clinical remission in patients with SEA [35].

The ZEPHYR‐4 study sought to illustrate the real‐world application of benralizumab in a demographically diverse asthma population, utilising an electronic health record (EHR) dataset representing > 90 million de‐identified patients in the USA [36]. A subgroup analysis of ZEPHYR‐4 aimed to describe clinical outcomes, based on key components of asthma remission. A subset of patients with ≥ 2 exacerbations during the 12‐month baseline period was also assessed. The proportion of patients achieving remission components after 12 months of benralizumab treatment was analysed.

## 3 Results

### 3.1 Achieving Remission in Clinical Trials and in the Real World

**FIGURE 1:**
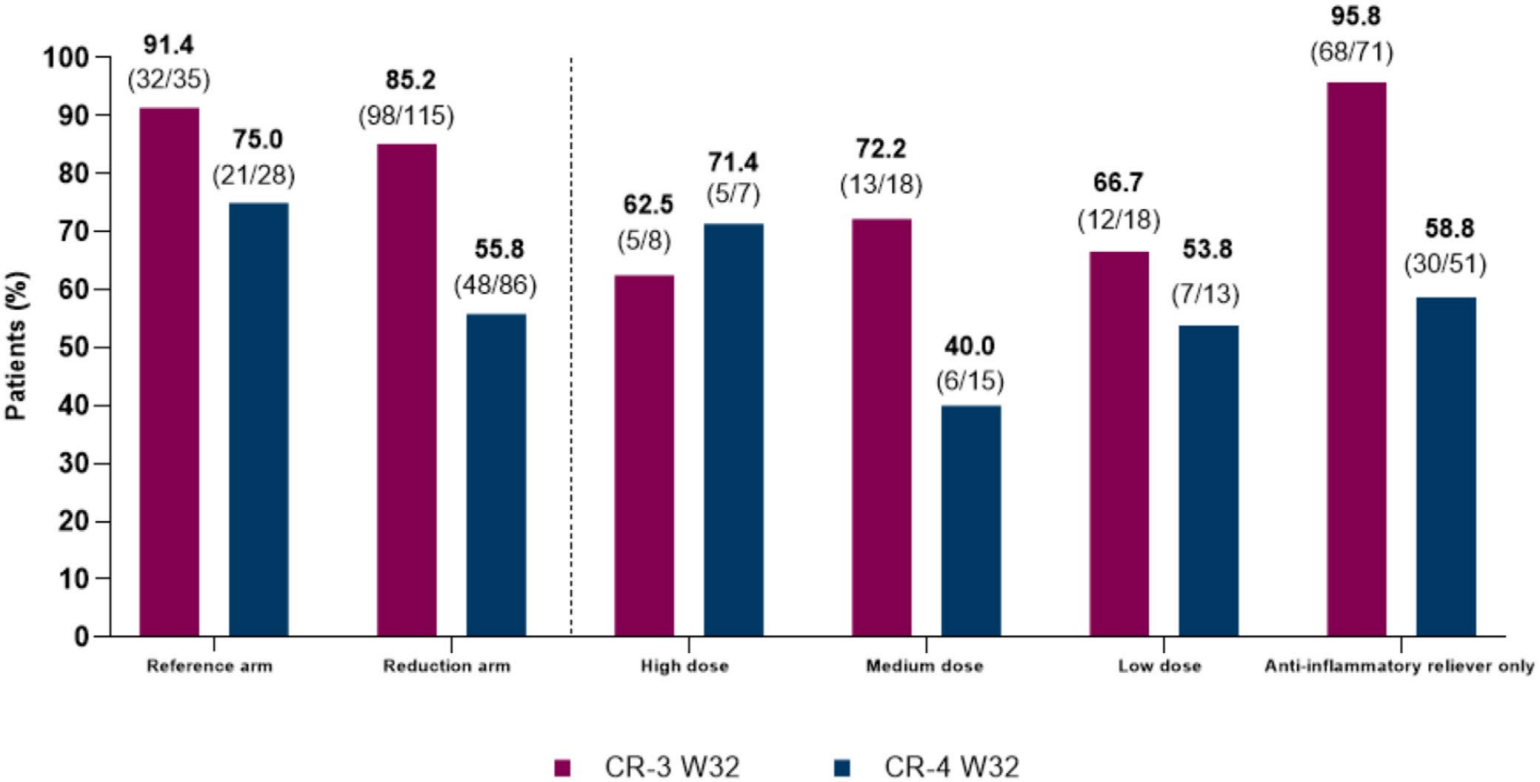
Patients^a^ with CR‐3 or CR‐4 at Week 32 in the SHAMAL subgroup analysis, by treatment arm and by ICS/formoterol dose. ^a^Percentages were calculated from patients with data available at Week 32, as denoted by numbers in parentheses. CR‐3 was a three‐component definition of remission, including no exacerbations, no OCS use and an ACQ‐5 score of < 1.5; CR‐4 additionally required a < 10% decrease in FEV1 from baseline. High dose: 1600 μg (budesonide 400 μg plus formoterol 12 μg per inhalation × 2 inhalations BID); medium dose: 800 μg (budesonide 200 μg plus formoterol 6 μg per inhalation × 2 inhalations BID); low dose: 400 μg (budesonide 200 μg plus formoterol 6 μg per inhalation × 1 inhalation BID); anti‐inflammatory reliever only: Budesonide 200 μg plus formoterol 6 μg per inhalation when needed. ACQ‐5, 5‐item Asthma Control Questionnaire; BID, twice daily; CR‐3, three‐component remission; CR‐4, four‐component remission; FEV_1_, forced expiratory volume in 1 s; OCS, oral corticosteroid.

In the *post hoc* analysis of SHAMAL, the majority of patients achieved remission regardless of background medication status. In the ICS/formoterol dose reduction arm, remission was achieved by 85.2% of patients for three‐component and 55.8% for four‐component clinical remission. Of patients with data available in the reduction arm, remission according to three‐ and four‐component criteria at Week 32 was achieved in 62.5% and 71.4% of patients (high dose [low patient numbers in this group skew the results]), 72.2% and 40.0% (medium dose), 66.7% and 53.8% (low dose), and 95.8% and 58.8% (anti‐inflammatory reliever only). In the reference arm (i.e., patients maintaining their ICS/formoterol dose), 91.4% and 75.0% of patients achieved remission at Week 32, respectively (Figure 1).

The post hoc analysis of AIP included 264 non‐OCS‐dependent patients receiving benralizumab. After 6 months, for evaluable patients, 105 (39.8%) patients achieved remission and 151 (57.2%) did not; 141 (53.6%) patients had a reduction in background medication use, including 74 (70.5%) patients with remission compared with 63 (41.7%) non‐remission patients (Table S3) After 18 months, 54/152 (35.5%) were in remission and 98/152 (64.5%) were not; the proportion of patients with background medication reductions per remission status was similar.

In an analysis of the retrospective XALOC‐1 RWE study over 12 months in 797 adults with SEA treated with benralizumab, 60% of patients achieved a composite of no exacerbations and no mOCS use. Furthermore, 43% met a remission definition of no exacerbations, no mOCS use and asthma symptom control.

#### 3.1.1 Long‐Term Remission Data

At the end of SIROCCO and CALIMA (12 months), 37.7% of patients achieved remission based on three‐component criteria, and 32.0% based on four‐component criteria. Following at least 12 months of continued treatment with benralizumab in BORA, 78.2% of patients who were in remission at the end of SIROCCO and CALIMA according to the three‐component criteria and 73.3% according to the four‐component criteria remained in remission at 24 months. Additionally, 31.7% and 25.8% of patients who did not achieve remission at the end of SIROCCO and CALIMA subsequently achieved three‐ and four‐component remission criteria, respectively, at 24 months after 12 months' additional treatment with benralizumab in BORA.

**FIGURE 2:**
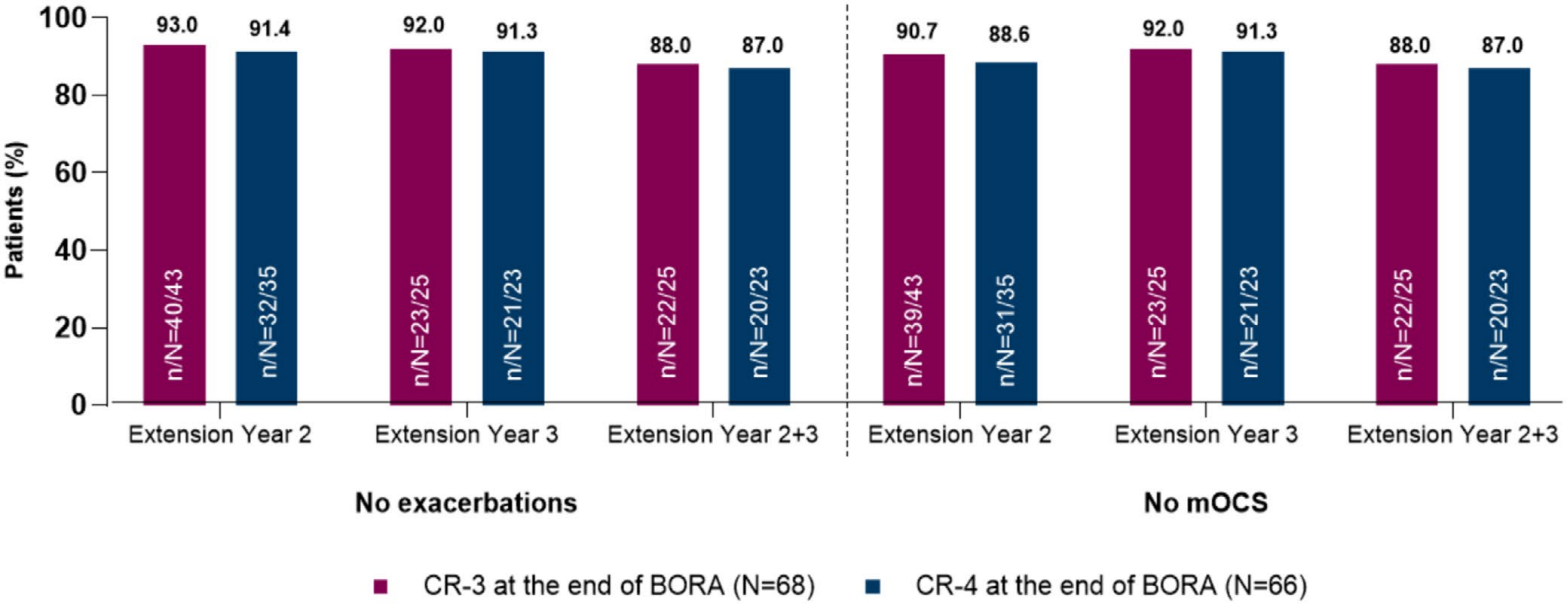
Asthma remission components achieved by MELTEMI patients at extension Years 2 and 3, by BORA remission status. Patients in BORA were considered to have achieved three‐component remission if they had no exacerbations, no mOCS use and ACQ‐6 score < 1.5; four‐component remission was considered to comprise three‐component remission and < 10% decrease from baseline in FEV_1_. ACQ‐6, 6‐item Asthma Control Questionnaire; CR‐3, three‐component remission; CR‐4, four‐component remission; FEV_1_, forced expiratory volume in one second; mOCS, maintenance oral corticosteroid.

The MELTEMI components of remission analysis included a total of 68 patients from MELTEMI Year 1 with remission data (three‐component criteria) for BORA and 66 patients with remission findings according to the four‐component criteria. Approximately 90% of patients with SEA who achieved either three‐ or four‐component remission status in the BORA study remained free from exacerbations and mOCS use after an additional year of benralizumab treatment (Figure 2). Achieving clinical remission with benralizumab in patients with SEA was predictive of long‐term freedom from exacerbations and mOCS use. Even among patients who did not achieve remission at Month 6 in the BORA study, more than 60% were free from exacerbations and mOCS use by the end of Year 1 in MELTEMI. Patients who did not initially achieve remission were still likely to derive additional benefit if they continued to receive benralizumab treatment.

#### 3.1.2 Predictors of Remission

**FIGURE 3:**
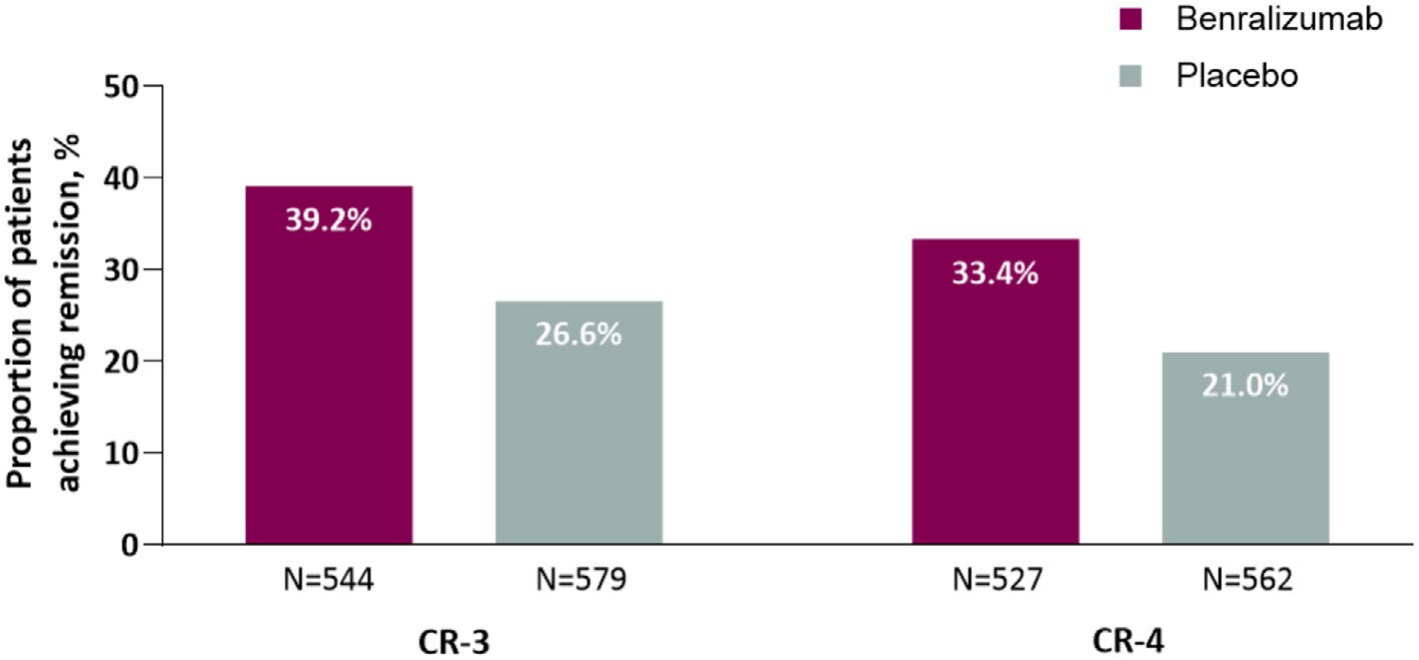
Proportion of patients achieving remission in SIROCCO and CALIMA. CR‐3 was a three‐component definition of remission that included no exacerbations, no use of OCS and an ACQ‐6 score of < 1.5; CR‐4 additionally required a ≤ 10% decrease in FEV_1_. ACQ‐6, 6‐item Asthma Control Questionnaire; CR‐3, three‐component remission; CR‐4, four‐component remission; FEV_1_, forced expiratory volume in one second; OCS, oral corticosteroid.

Among 1123 patients from the SIROCCO/CALIMA *post hoc* analysis, 39.2% and 26.6% achieved remission on benralizumab and placebo, respectively, according to the three‐component definition, and 33.4% and 21.0% achieved remission according to the four‐component definition (Figure 3) Compared with non‐remission patients, those with remission had higher baseline median blood eosinophil counts, were more likely to have an FEV_1_ ≥ 65% predicted, were less likely to have had greater than two exacerbations within 12 months of baseline and had lower baseline ACQ‐6 mean scores (Table S4). Baseline characteristics for patients achieving three‐ and four‐component remission criteria in SIROCCO and CALIMA from the BORA *post hoc* analysis are shown in Table S5

**FIGURE 4:**
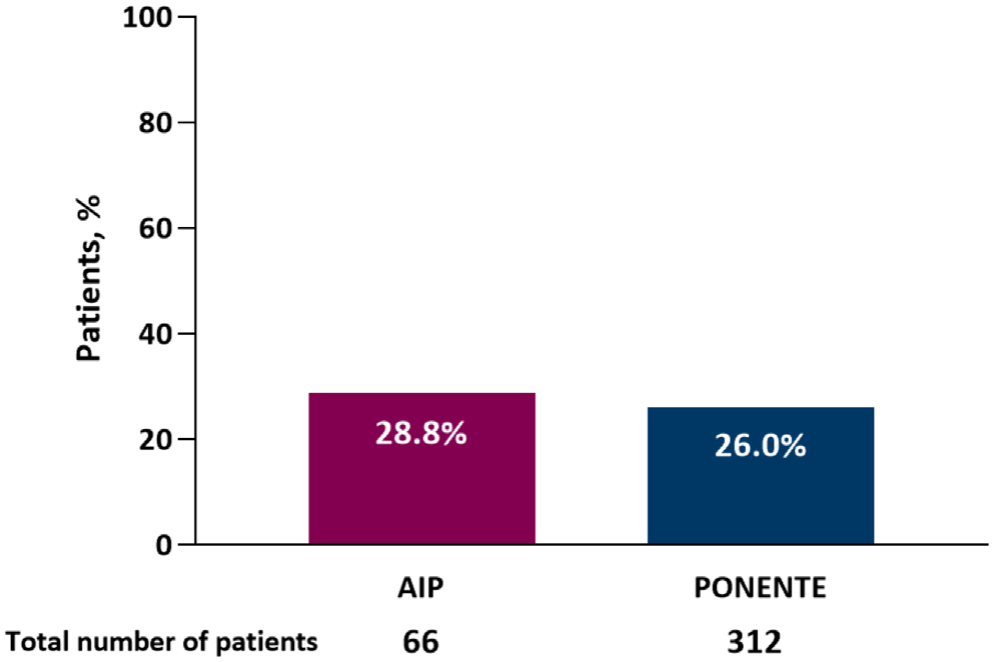
Proportion of patients achieving remission in the *post hoc* analysis of AIP and PONENTE. Remission was a composite measure defined as no exacerbations, no use of OCS and an ACQ‐6 score < 1.5. Lung function was not included in the definition because it was not measured in patients who participated in the PONENTE study. ACQ‐6, 6‐item Asthma Control Questionnaire; AIP, ANDHI‐In practice; OCS, oral corticosteroid.

In the *post hoc* analysis of AIP and PONENTE, 28.8% of the 66 patients in the AIP cohort treated with mOCS attained remission and 26.0% of 312 evaluable patients achieved remission in the PONENTE cohort (Figure 4). Patients achieving remission in both AIP and PONENTE exhibited a shorter mean time since their asthma diagnosis compared with patients who did not achieve remission (Table S6). Additionally, the mean age at asthma onset for patients with remission was higher, and they exhibited lower ACQ‐6 mean scores compared with non‐remission patients. In those patients receiving mOCS, the levels of baseline blood eosinophil counts do not seem to predict remission (Table S6)

#### 3.1.3 Effects of Comorbidities in Clinical Trials and in the Real World

In the SIROCCO/CALIMA *post hoc* analysis, compared with the overall population, patients with a history of nasal polyps were more likely to achieve remission with benralizumab, whereas this was not observed in patients who received placebo (Table S4)

**FIGURE 5:**
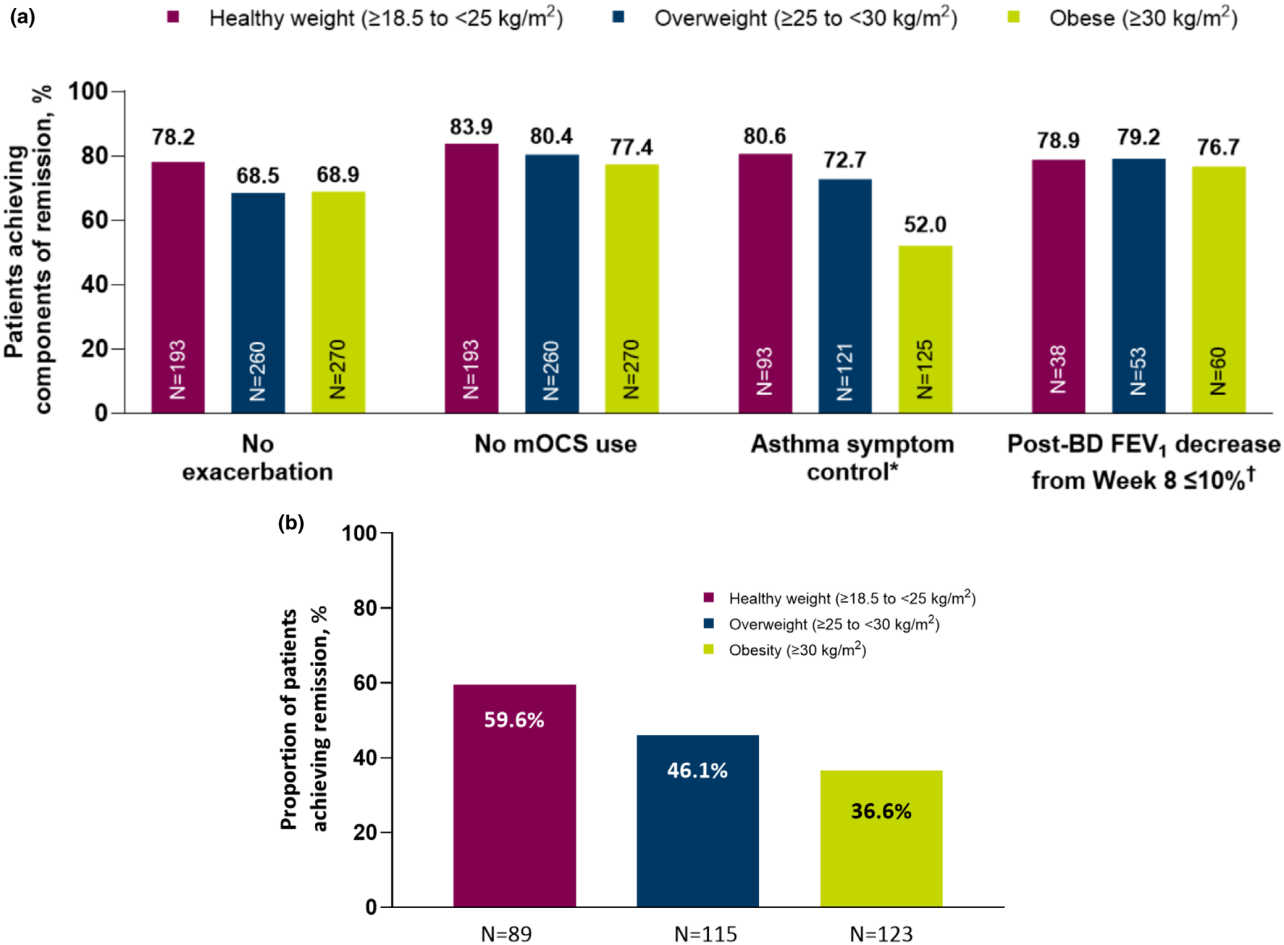
(a) Components of remission observed at Week 48 in the XALOC‐1 integrated analysis, by BMI status. Remission encompassed the following four key outcomes, evaluated individually or as a composite: no exacerbations, no mOCS use, asthma symptom control and no deterioration of lung function. ^*^Defined as an ACQ‐6 score < 1.5 or ACT score ≥ 16. ^†^These data should be interpreted with caution due to the small patient numbers and uneven distribution of data between BMI status categories. (b) Proportion of patients achieving three‐component clinical remission in the XALOC‐1 sub‐analysis, by BMI status. CR‐3 was a three‐component definition of remission based on no exacerbations, no mOCS use and asthma symptom control (ACQ‐6 and ACT score). Exacerbations were assessed throughout 48 weeks of benralizumab treatment. mOCS use, asthma symptoms and FEV_1_ were assessed at Week 48. ACQ‐6, 6‐item Asthma Control Questionnaire; ACT, Asthma Control Test; BD, bronchodilator; BMI, body mass index; CR‐3, three‐component remission; FEV_1_, forced expiratory volume in 1 s; mOCS, maintenance oral corticosteroid.

In the sub‐analysis of 797 patients from the XALOC‐1 study, the exacerbation and mOCS use components of remission were not dramatically affected by BMI; however, patients with a lower BMI were more likely to achieve asthma symptom control and therefore remission (Figure 5a). The three‐component definition of remission, that is no exacerbations, no mOCS use and asthma symptom control, was achieved by 59.6%, 46.1% and 36.6% of patients who were of normal weight, overweight and obese, respectively (Figure 5b).

The BPAP study found that patients with comorbidities, including obesity, were less likely to achieve clinical remission with benralizumab. Specifically, BMI and mOCS use were negatively associated with clinical remission at 1 and 2 years of follow‐up [35].

### 3.2 Achieving Remission in the Real World and Stratification by Blood Eosinophil Count and Total Serum IgE

**FIGURE 6:**
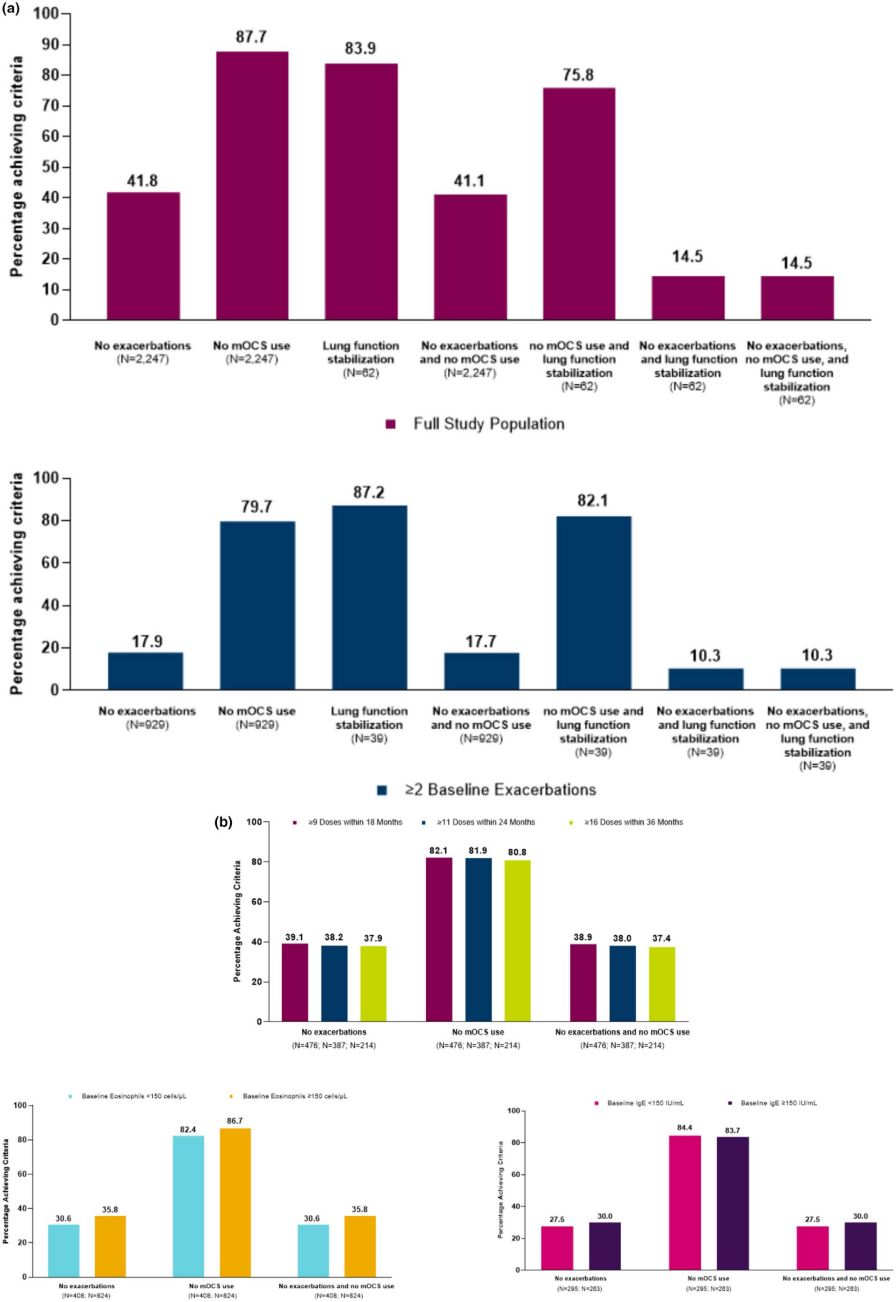
(a) Asthma remission components at 12 months post‐benralizumab initiation in the ZEPHYR‐4 analysis. Remission components included no exacerbations, no mOCS use and lung function stabilisation, and excluded ACT score due to data unavailability. (b) Subgroup analysis of asthma remission components according to long‐term benralizumab use, blood eosinophil count and serum IgE in the ZEPHYR‐4 analysis. Remission components included no exacerbations, no mOCS use and lung function stabilisation, and excluded ACT score due to data unavailability. ACT, Asthma Control Test; IgE, immunoglobulin E; mOCS, maintenance oral corticosteroid.

Among the 2895 patients included in the ZEPHYR‐4 analysis, a subgroup of 1275 was identified with ≥ 2 baseline exacerbations. In the full study population and the subgroup of patients with greater than or equals to two baseline exacerbations, 41.8% and 17.9% were free of exacerbations, whereas 87.7% and 79.7% reported no mOCS use, and 83.9% and 87.2% achieved lung function stabilisation after 12 months of treatment with benralizumab, respectively (Figure 6a). Additionally, 14.5% and 10.3% of patients achieved all three components of remission (the number of patients with FEV_1_ measurements during baseline and follow‐up was too low for meaningful analysis). Subgroup analysis findings for remission components generally aligned with those of the full study population. However, the baseline blood eosinophil analysis indicated a slightly stronger response among individuals with ≥ 150 cells/μL compared with those with < 150 cells/μL, whereas total serum IgE levels did not appear to impact the response (Figure 6b).

## 4 Discussion

The advent of biologics has made it possible for remission to become a realistic treatment goal for patients with SEA, and by consolidating *post hoc* remission data from clinical trials and RWE studies of benralizumab we have gained further insights and learnings in this regard. For example, findings consistently indicate that patients with less severe disease, and when treated earlier, are more likely to achieve remission with benralizumab [27, 28, 31, 32]. Patients with higher blood eosinophil counts at baseline are also more likely to achieve remission after treatment with benralizumab, consistent with its mechanism of action [27, 28]. Furthermore, RWE studies have shown that patients with a lower disease burden have a higher likelihood of reaching remission, indicating that earlier treatment intervention with benralizumab may be beneficial.

Understanding the impact of pulmonary and extrapulmonary comorbidities on asthma symptoms, and, through that, the ability to reach predefined remission thresholds for symptom control, is crucial for optimising treatment strategies and improving patient outcomes. In particular, obesity is a common comorbidity in asthma and has been shown to negatively impact asthma symptom control and create a greater reliance on OCS; consequently, this is an important subgroup in whom the assessment of clinical remission of asthma can be challenging [35]. Additionally, current tools like the ACQ may have limitations in distinguishing between asthma symptoms and those arising from comorbidities.

The availability of biologic treatments for SEA has led to an interest in whether background therapies can be reduced, and what impact this might have on remission. The GINA guidelines, now updated according to SHAMAL study findings, recommend the reduction of background medications when asthma is controlled on a biologic [24]. Both the SHAMAL study and the Phase 3b AIP substudy show that the majority of patients achieved remission with benralizumab despite reductions in background medications [26, 31]. In the *post hoc* analysis of the SHAMAL study, among patients who reduced their ICS/formoterol dose, particularly those who tapered to anti‐inflammatory reliever only, most achieved three‐component clinical remission, but fewer met the four‐component criteria. This difference is likely due to the lung function component of the four‐component criteria, as down‐titration of ICS affected lung function in some patients, limiting the ability to achieve the stricter four‐component remission [26].

Once remission is achieved with benralizumab, most patients appear to experience long‐term benefits when treatment is continued. This has been demonstrated in both extension studies (BORA and MELTEMI) and in real‐world studies like XALOC‐1 [33]. Moreover, some patients may require prolonged treatment to achieve remission. For example, many patients who did not achieve remission by the end of the 12‐month SIROCCO and CALIMA studies went on to achieve components of remission by the end of the 24‐month BORA study [37]. Even those who did not achieve remission during the BORA study may still achieve components of remission in the longer term, as suggested by findings from the post hoc analysis of the MELTEMI study. Physicians should, therefore, not rush to switch biologic therapy in cases where remission is not seen after 1 year as patients may well derive additional benefit after further treatment with benralizumab.

The real‐world studies have shown that remission rates with benralizumab are consistent with, and in some cases higher than, those observed in clinical trials. However, it is important to note that lung function is not always available in RWE studies and so remission rates may be increased in comparison with randomised controlled trials which also evaluate lung function. In randomised controlled trials, participants often have impaired lung function at baseline (as a requirement for trial eligibility), which can make achieving more ambitious lung function goals, such as FEV_1_ ≥ 80%, difficult [38].

The definition of remission in asthma, particularly with regard to lung function criteria, remains a subject of debate [39]. The *post hoc* analyses reviewed here applied different criteria for remission, often including a ≤ 10% decrease in FEV_1_ as part of the more stringent four‐component remission criteria (Table S1) However, it is important to recognise that for many patients with moderate‐to‐severe asthma, especially those on lower‐dose ICS or anti‐inflammatory relievers only, achieving optimal lung function may not be a feasible goal, even if other remission criteria are met [26]. Including a ≤ 10% decrease in FEV_1_ as part of the four‐component remission criteria allows for the inclusion of patients with impaired lung function who still demonstrate overall clinical benefits. The adoption of more lenient thresholds helps ensure that a broader population of patients, who may still benefit from treatment despite suboptimal lung function, is included in remission analyses.

‘Treat‐to‐target’ (TTT) is a therapeutic approach where treatment decisions are aimed at reaching and maintaining predefined clinical goals [40]. The concept of TTT is gaining traction in the management of SEA, mirroring its emergence in the management of other chronic conditions, such as rheumatoid arthritis [41]. When applying this TTT approach to asthma, it has been suggested that the primary aim could centre on achieving remission, with specific objectives, including eliminating symptoms and exacerbation risk, preventing airway remodelling and restoring lung function to normalcy [41].

In the future, it will be of interest to further develop the concept of remission by incorporating both clinical *and* biological remission. Whereas clinical remission focuses on exacerbations, mOCS use, symptom control and lung function optimisation/stabilisation, biological remission pertains to the absence of relevant disease biomarkers that are usually assessed through laboratory tests. Biomarkers may offer further insight into the state of disease activity, providing a deeper understanding beyond clinical observations alone [42]. This might be particularly crucial when it comes to stepping down treatment after remission has been achieved for a long period, for instance by reducing background medication (e.g., ICS). Parallel studies in the gastroenterology field suggest that relying solely on clinical remission as the only treatment endpoint may be inadequate, and the inclusion of biological remission is recommended as a treatment target [42]. It will also be of interest to investigate potential impacts of remission that exist beyond symptom control and OCS/exacerbation reduction. These may include improvements in quality of life and other patient‐reported outcomes (e.g., treatment satisfaction), a decrease in emergency visits and hospitalisations, prevention of accelerated lung function decline, reduced healthcare costs, a reduction in background medication, as well as a positive impact on comorbidities [10].

## 5 Conclusions

In summary, this article provides valuable insight concerning the feasibility of achieving remission with benralizumab in patients with SEA. Although some patients may attain remission within 12 months, others may require a longer duration of treatment with benralizumab to derive the full benefit of treatment. Our findings may therefore guide the future direction of remission research, as part of a new era of TTT where prioritising early treatment to maximise patient outcomes and attain remission stands as the ultimate therapeutic goal. In this regard, standardising the definition of remission and developing new tools to measure remission would be of value.

## Author Contributions

All authors contributed to the intellectual input and critical revision of the manuscript. Each author reviewed the drafts for content accuracy and provided feedback. All authors have read and approved the final version of the manuscript.

## Conflicts of Interest

R.L. has received research grants from AstraZeneca, Chiesi, GSK, and Sanofi, and consultancy fees and speakers' fees from AstraZeneca, GSK, and Sanofi. M.L. has received grants/research support from AstraZeneca, DFG, and GSK, and honoraria or consultation fees from ALK, Abelló, Allergopharma, Apontis, AstraZeneca, Bencard, Berlin‐Chemie, Boehringer Ingelheim, Boston Scientific, Chiesi, GSK, Janssen, MSD, Mundipharma, Novartis, Nycomed/Takeda, Sanofi, Stallergens, Teva and UCB, D.J.J. has received consultancy fees and speakers' fees from AstraZeneca, Boehringer Ingelheim, GSK, Sanofi Regeneron and Chiesi, and research grants from AstraZeneca. A.M.‐G., A.S. and D.C. are all employees of AstraZeneca and may own stock in the company. F.C.L.H. has received speaker fees from AstraZeneca as well as honoraria or consultation fees from AstraZeneca, GSK, Sanofi, Teva and Genentech. S.K. has received consultancy fees and speakers' fees from AstraZeneca, Chiesi, GSK, and Sanofi, and research grants from AstraZeneca and GSK.

## Supporting information


Data S1.

## Data Availability

Data underlying the findings described in this manuscript may be obtained in accordance with AstraZeneca's data sharing policy described at https://astrazenecagrouptrials.pharmacm.com/ST/Submission/Disclosure. Data described in this manuscript have previously only been presented at congresses.
