# Supplementary material for: Advancing Remission in Severe Asthma With Benralizumab: Latest Findings, Current Perspectives and Future Direction
Source: Clin Exp Allergy. 2025 May 30;55(7):521–31. doi: 10.1111/cea.70083 (PMC12221862; doi:10.1111/cea.70083)
Supplement: Supplementary file 1 — Data S1. [file CEA-55-521-s001.docx]

**Supplementary Material**

**Supplementary Table 1. Summary of asthma remission methods and sub-analyses from interventional studies**

| Study ID | Study type | Study population in *post-hoc* analyses | Inclusion/exclusion criteria for *post-hoc* analyses | Study duration | Remission component assessed in *post-hoc* analyses | Remission definition(s) in *post-hoc* analyses |
| --- | --- | --- | --- | --- | --- | --- |
| SIROCCO  (NCT01928771) **[1]** | RCT; double-blind, parallel-group, placebo-controlled Phase 3 | Patients with uncontrolled SEA aged 12–75 years | - Adults receiving medium-/high-dose ICS/LABA - Completion of treatment in SIROCCO or CALIMA studies - Patients with mOCS use were excluded in this analysis | 48 weeks | Baseline characteristics in patients who did or did not achieve remission after 12 months | CR-3 was defined as no exacerbations, no OCS, and an ACQ-6 score of <1.5; CR-4 additionally required a ≤10% decrease in FEV_1_ |
| CALIMA  (NCT01914757) **[2]** | RCT; double-blind, parallel-group, placebo-controlled, Phase 3 |  |  | 56 weeks |  |  |
| ANDHI-In Practice (AIP)  (NCT03170271) **[3]** | RCT; 56-week single-arm, open-label extension of the 24-week Phase 3b, double blind, placebo-controlled ANDHI study | Patients with uncontrolled SEA aged 18–75 years | - Patients had ≥2 asthma exacerbations in the previous year despite receiving medium- or high-dose ICS plus another controller - Pre-BD FEV_1_ was <80% predicted, and the ACQ-6 score was ≥1.5 - bEOS count was ≥300 cells/μL or ≥150 cells/μL with 1 of the following: Maintenance OCS, history of nasal polyps, ≥3 exacerbations in prior year, FVC of <65%, or asthma diagnosis at age ≥18 years | 12 months | A composite definition of remission was used to evaluate remission rates after 6 (the end of ANDHI) and 18 months (the end of AIP) of treatment with benralizumab and the proportion of patients who reduced background medications after 18 months  Baseline characteristics and remission rates among patients in AIP and PONENTE who were OCS dependent, defined as daily OCS dose ≥5 mg for ≥3 months | Patients were considered to be in remission if they met all three components: no exacerbations, no OCS use, and an ACQ-6 score of <1.5 |
| PONENTE (NCT03557307) **[4]** | RCT; single-arm, open-label, Phase 3b | Patients with uncontrolled SEA aged ≥18 years | - Patients received high-dose ICS and LABA for ≥6 months preceding enrolment - Patients received mOCS for ≥3 months - bEOS count was ≥150 cells/μL at enrolment or ≥300 cells/μL in the past 12 months | 12 months | Baseline characteristics and remission rates among patients in AIP and PONENTE who were OCS dependent, defined as daily OCS dose ≥5 mg for ≥3 months | Remission was defined as no exacerbations, no OCS, and an ACQ-6 score of <1.5 after 18 (the end of ANDHI-IP) or 12 (the end of PONENTE) months of treatment with benralizumab |
| SHAMAL (NCT04159519) **[5]** | RCT; open-label, parallel group Phase 4 | Patients with controlled SEA aged ≥18 years | - Current maintenance treatment with high-dose ICS/FORM - Patients received ≥3 consecutive doses of benralizumab prior to Visit 1 (screening) - Controlled asthma (ACQ-5 score <1.5 at Visit 1) | 52-56 weeks | Maintenance ICS/LABA dose reduction and the associated remission status at Week 32 | Remission was defined as meeting either of two criteria sets; CR-3: no exacerbations, no OCS use, and an ACQ-5 score of <1.5; CR-4 additionally required a <10% decrease in FEV_1_ from baseline |
| BORA (NCT02258542) **[6]** | Longitudinal; double-blind, parallel group, Phase 3 extension | Patients with uncontrolled SEA aged 12–75 years | - Patients had no mOCS use at baseline in the preceding SIROCCO and CALIMA studies - Patients transitioned into the BORA study for ≥12 additional months | 1–2 years  (2–3 years total benralizumab exposure) | Percentage and baseline characteristics of patients achieving durable remission | Patients were assessed using two definitions of asthma remission: CR-3, consisting of no exacerbations, no mOCS use, and an ACQ-6 score of <1.5, and CR-4, consisting of CR-3 and a ≤10% decline from Week 8 FEV_1_ |
| MELTEMI (NCT02808819) **[7]** | Longitudinal; open-label, Phase 3 safety extension | Patients with uncontrolled SEA aged 18–75 years | - Patients with SEA who had no maintenance OCS use at baseline in SIROCCO/CALIMA and remission status at Month 6 in the BORA study - Received benralizumab 30 mg Q8W for 12 months in predecessor studies and for at least 6 months in the BORA study - Available data for use of maintenance OCS and exacerbations after 1 year in MELTEMI | 2 years  (4–5 years total benralizumab exposure) | Longer-term outcomes in patients who achieved remission | The proportion of patients experiencing no exacerbations or no mOCS use were examined after the first year of MELTEMI. The study did not capture ACQ-6 or FEV_1_ outcomes |

ACQ-5, 5-item Asthma Control Questionnaire; ACQ-6, 6-item Asthma Control Questionnaire; ACT, Asthma Control Test; AIP, ANDHI-In Practice; BD, bronchodilator; bEOS, blood eosinophil; BMI, body mass index; CR-3, 3-component clinical remission; CR-4, 4-component remission; FEV_1_, forced expiratory volume in one second; FORM, formoterol; ICS, Inhaled corticosteroid; LABA, long-acting β_2_-agonist; (m)OCS, (maintenance) oral corticosteroid; RCT, randomized controlled trial; SEA, severe eosinophilic asthma.

**Supplementary Table 2. Summary of asthma remission methods and analyses from real-world evidence studies**

| Study ID | Study type | Study population | Study duration | Remission component assessed | Remission definition(s) |
| --- | --- | --- | --- | --- | --- |
| XALOC-1 **[8]** | RWE study; multinational, retrospective | Patients with SEA aged ≥18 years | Up to 96 weeks | Remission in patients with/without prior biologic experience, and by baseline characteristics | The analysis evaluated the percentage of patients meeting the individual as well as the composite components of remission (no exacerbations, no mOCS use, and asthma symptom control [ACT score ≥16 or ACQ-6 <1.5]) at Weeks 0, 48, and 96  As a sensitivity analysis, a stricter definition of remission was also used, requiring an ACT score of ≥20 or an ACQ-6 score of ≤0.75 (indicating well-controlled asthma) |
|  |  |  |  | The impact of BMI on remission | Remission criteria encompassed the following four key outcomes, evaluated individually or as a composite: no exacerbations, no mOCS use, asthma symptom control, and lung function  CR-3 was based on no exacerbations, no mOCS use, and asthma symptom control |
| ZEPHYR-4 **[9]** | RWE study; retrospective, observational | Patients initiating benralizumab in real-world US practice using EHR data | 12 months post-benralizumab initiation | Clinical outcomes, based on key components of remission | Remission components included no exacerbations, no mOCS use, and lung function stabilization, and excluded ACT due to data unavailability |

ACQ-6, 6-item Asthma Control Questionnaire; ACT, Asthma Control Test; BMI, body mass index; CR-3, 3-component clinical remission; EHR, electronic health record; mOCS, oral corticosteroid; RWE, real-world evidence; SEA, severe eosinophilic asthma.

**Supplementary Table 3. Proportion of patients with background medication reductions at the end of AIP (18 months) according to remission status either: at the end of ANDHI (6 months) or the end of AIP (18 months) in the AIP Phase 3b substudy**

|  | According to remission status at the end of ANDHI (6 months) | | According to remission status at the end of AIP (18 months) | |
| --- | --- | --- | --- | --- |
|  | N | Proportion of patients with background medication reductions at the end of AIP, % | N | Proportion of patients with background medication reductions at the end of AIP, % |
| Overall | 264 | 53.6 | 152 | 60.5 |
| Clinical remission | 106 | 70.5 | 54 | 79.6 |
| Non-remission | 151 | 41.7 | 98 | 50.0 |

**Supplementary Table 4. Patient demographics and clinical characteristics from the SIROCCO/CALIMA *post-hoc* remission analysis**

|  | CR-3 | | | | CR-4 | | | |
| --- | --- | --- | --- | --- | --- | --- | --- | --- |
|  | Benralizumab | | Placebo | | Benralizumab | | Placebo | |
|  | Remission (N=213) | Non-remission (N=331) | Remission (N=154) | Non-remission (N=425) | Remission (N=176) | Non-remission (N=351) | Remission (N=118) | Non-remission (N=444) |
| Median age, years (range) | 50.0 (12–74) | 53.0 (12–74) | 50.5 (12–75) | 52.0 (12–75) | 50.5 (12–74) | 52.0 (12–74) | 52.0 (12–75) | 52.0 (12–75) |
| Sex, female, n (%) | 121 (56.8) | 218 (65.9) | 91 (59.1) | 277 (65.2) | 100 (56.8) | 228 (65.0) | 70 (59.3) | 287 (64.6) |
| Local baseline bEOS count cells/µL, median | 412.0 | 365.0 | 402.0 | 360.0 | 388.0 | 371.0 | 419.0 | 360.0 |
| (range) | (0–2095) | (0–3100) | (10–3640) | (0–2610) | (0–2095) | (0–3100) | (100–3640) | (0–2610) |
| Local baseline bEOS ≥300 cells/µL, n (%) | 152 (72.0) | 208 (63.8) | 113 (73.9) | 275 (65.3) | 125 (71.0) | 222 (63.2) | 89 (75.4) | 288 (64.9) |
| Median time since asthma diagnosis, years (range) | 14.6 (1–63) | 15.3 (1–65) | 13.6 (1–72) | 15.6 (1–70) | 14.7 (1–63) | 15.3 (1–65) | 13.4 (1–72) | 15.7 (1–70) |
| Median age at asthma onset, years (range) | 30.0 (0–65) | 33.3 (0–72) | 29.4 (0–67) | 32.6 (0–72) | 30.5 (0–65) | 33.0 (0–72) | 32.6 (0–66) | 32.0 (0–72) |
| Total IgE, IU/mL, median (range) | 166.3 | 206.1 | 185.3 | 179.7 | 165.1 | 206.3 | 206.1 | 178.8 |
|  | (4–5782) | (2–12754) | (5–10,029) | (2–17,317) | (4–5590) | (2–12,754) | (5–10,029) | (2–17,317) |
| Phadiatop positive at baseline, n (%) | N=211 | N=323 | N=153 | N=418 | N=174 | N=344 | N=117 | N=438 |
|  | 136 (64.5) | 209 (64.7) | 98 (64.1) | 246 (58.9) | 114 (64.8) | 222 (63.2) | 79 (66.9) | 258 (58.1) |
| Median ACQ-6 score (range) | 2.5 (0–5) | 2.8 (1–6) | 2.5 (1–6) | 2.8 (1–6) | 2.5 (0–5) | 2.8 (1–6) | 2.5 (1–6) | 2.8 (1–6) |
| History of nasal polyps, n (%) | 42 (19.7) | 38 (11.5) | 22 (14.3) | 76 (17.9) | 36 (20.5) | 42 (12.0) | 16 (13.6) | 81 (18.2) |
| FEV_1_, n (%) |  |  |  |  |  |  |  |  |
| <65% predicted normal | 130 (61.3) | 233 (70.6) | 91 (59.1) | 274 (66.2) | 106 (60.2) | 247 (70.4) | 71 (60.2) | 289 (65.1) |
| ≥65% predicted normal | 82 (38.7) | 97 (29.4) | 63 (40.9) | 140 (33.8) | 69 (39.2) | 103 (29.3) | 47 (39.8) | 147 (33.1) |
| Exacerbations at baseline, n (%) |  |  |  |  |  |  |  |  |
| 2 | 151 (70.9) | 216 (65.3) | 114 (74.0) | 260 (61.2) | 125 (71.0) | 230 (65.5) | 90 (76.3) | 275 (61.9) |
| >2 | 61 (28.6) | 115 (34.7) | 40 (26.0) | 165 (38.8) | 50 (28.4) | 121 (34.5) | 28 (23.7) | 169 (38.1) |

CR-3 was a 3-component definition of remission that included no exacerbations, no use of oral corticosteroids, and an ACQ-6 score of <1.5; CR-4 additionally required a ≤10% decrease in FEV_1_.

ACQ-6, 6-item Asthma Control Questionnaire; bEOS, blood eosinophil; FEV_1_, forced expiratory volume in one second; IgE, immunoglobulin E; IU, international unit; SD, standard deviation.

**Supplementary Table 5. Baseline characteristics for patients achieving remission in SIROCCO and CALIMA from the BORA *post-hoc* analysis**

| Characteristic | CR-3 (N=334) | | CR-4 (N=325) | |
| --- | --- | --- | --- | --- |
|  | Remission (N=126) | Non-remission (N=208) | Remission (N=104) | Non-remission (N=221) |
| Median age, years (range) | 50.0 (19–74) | 54.0 (21–74) | 51.0 (19–74) | 54.0 (21–74) |
| Female sex, n (%) | 76 (60.3) | 128 (61.5) | 62 (59.6) | 138 (62.4) |
| Baseline bEOS count cells/µL, median (range) | N=125 427 (0–1860) | N=204 350 (0–3100) | 425 (0–1360) | N=216 350 (0–3100) |
| Median time since asthma diagnosis, years (range) | 15.9 (1–58) | 15.4 (1–58) | 16.0 (1–57) | 15.5 (1–58) |
| Median ACQ-6 score (range) | 2.4 (0–5) | 2.8 (1–6) | 2.3 (0–5) | 2.8 (1–6) |
| Baseline FEV_1_ ≥65% predicted normal, n (%) | 52 (41.3) | 48 (23.1) | 45 (43.3) | 50 (22.6) |

CR-3 was a 3-component definition of remission that included no exacerbations, no use of oral corticosteroids, and an ACQ-6 score of <1.5; CR-4 additionally required a ≤10% decrease in FEV_1_.

ACQ-6, 6-item Asthma Control Questionnaire; bEOS, blood eosinophil; FEV_1_, forced expiratory volume in one second.

**Supplementary Table 6. Patient demographics and clinical characteristics from the AIP and PONENTE *post-hoc* remission analysis**

|  | AIP (N=66) | | PONENTE (N=312) | |
| --- | --- | --- | --- | --- |
|  | Remission  (N=19) | Non-remission (N=47) | Remission (N=81) | Non-remission (N=231) |
| Median age, years (range) | 61.0 (36–71) | 54.0 (24–70) | 53.0 (22–75) | 54.0 (20–81) |
| Female sex, n (%) | 12 (63.2) | 33 (70.2) | 47 (58.0) | 150 (64.9) |
| bEOS count, median (range) | 410  (200–1040) | 400  (150–1810) | 190  (60–900) | 220  (30–1790) |
| bEOS ≥300 cells/µL, n (%) | 14 (73.7) | 32 (68.1) | 16 (20.0) | 81 (35.5) |
| Median time since asthma diagnosis, years (range) | 15.5 (3–42) | 21.4 (1–69) | 15.1 (1–68) | 20.0 (0–73) |
| Median age at asthma onset, years (range) | 39.0 (19–68) | 29.0 (0–58) | 39.0 (0–68) | 29.0 (0–74) |
| Total IgE, IU/mL, median (range) | 234.7  (8–3923) | 85.7  (2–893) | 93.0  (3–4167) | 131.4 (2–17,841) |
| Phadiatop positive at baseline, n (%) | 8 (44.4) | 20 (44.4) | 37 (46.3) | 107 (46.7) |
| Median ACQ-6 score (range) | 2.7 (2–4) | 3.2 (2–5) | 1.5 (0–6) | 2.5 (0–6) |
| History of nasal polyps, n (%) | 9 (47.4) | 14 (29.8) | 23 (28.4) | 77 (33.3) |
| OCS use at baseline, n (%)  Median OCS dose at baseline, mg (range) | 17 (89.5)  5.0 (2–20) | 45 (95.7)  10.0 (1–20) | 81 (100)  10.0 (5–50) | 231 (100)  10.0 (5–60) |
| FVC, n (%)  <65% predicted normal  ≥65% predicted normal | 1 (5.3)  18 (94.7) | 13 (27.7)  34 (72.3) | -  - | -  - |
| Exacerbations at baseline, n (%)  <2  2  >2 | -  8 (42.1)  11 (57.9) | -  17 (36.2)  30 (63.8) | 37 (45.7)  14 (17.3)  30 (37.0) | 78 (33.8)  44 (19.0)  109 (47.2) |

ACQ-6, 6-item Asthma Control Questionnaire; AIP, ANDHI-In Practice; bEOS, blood eosinophil; FVC, forced vital capacity; IgE, immunoglobulin E; OCS, oral corticosteroid.

**References**

1. Bleecker ER, FitzGerald JM, Chanez P, et al. Efficacy and safety of benralizumab for patients with severe asthma uncontrolled with high-dosage inhaled corticosteroids and long-acting β. *Lancet* 2016; 388: 2115-2127. DOI: 10.1016/s0140-6736(16)31324-1.

2. FitzGerald JM, Bleecker ER, Nair P, et al. Benralizumab, an anti-interleukin-5 receptor α monoclonal antibody, as add-on treatment for patients with severe, uncontrolled, eosinophilic asthma (CALIMA): A randomised, double-blind, placebo-controlled phase 3 trial. *Lancet* 2016; 388: 2128-2141. DOI: 10.1016/s0140-6736(16)31322-8.

3. Louis R, Harrison TW, Chanez P, et al. Severe asthma standard-of-care background medication reduction with benralizumab: ANDHI in Practice substudy. *J Allergy Clin Immunol Pract* 2023; 11: 1759-1770. DOI: 10.1016/j.jaip.2023.03.009.

4. Menzies-Gow A, Gurnell M, Heaney LG, et al. Oral corticosteroid elimination via a personalised reduction algorithm in adults with severe, eosinophilic asthma treated with benralizumab (PONENTE): A multicentre, open-label, single-arm study. *Lancet Respir Med* 2022; 10: 47-58. DOI: 10.1016/s2213-2600(21)00352-0.

5. Jackson DJ, Heaney LG, Humbert M, et al. Reduction of daily maintenance inhaled corticosteroids in patients with severe eosinophilic asthma treated with benralizumab (SHAMAL): A randomised, multicentre, open-label, phase 4 study. *Lancet* 2024; 403: 271-281. DOI: 10.1016/S0140-6736(23)02284-5.

6. Busse WW, Bleecker ER, FitzGerald JM, et al. Long-term safety and efficacy of benralizumab in patients with severe, uncontrolled asthma: 1-year results from the BORA phase 3 extension trial. *Lancet Respir Med* 2019; 7: 46-59. DOI: 10.1016/S2213-2600(18)30406-5.

7. Korn S, Bourdin A, Chupp G, et al. Integrated safety and efficacy among patients receiving benralizumab for up to 5 years. *J Allergy Clin Immunol Pract* 2021; 9: 4381-4392. DOI: 10.1016/j.jaip.2021.07.058.

8. Pelaia G, Jackson DJ, Nair P, et al. Clinical remission over 2 years with benralizumab in severe eosinophilic asthma: Real-world XALOC-1 program. *J Allergy Clin Immunol Pract* 2024 (in press).

9. Carstens D, Ojeranti D, Chung Y, Chan K and Dhopeshwarkar N. Heterogeneous characteristics of severe asthma patients initiating benralizumab in a real-world setting: The ZEPHYR-4 study. *Ann Allergy Asthma Immunol* 2023; 131: S51-S52. DOI: 10.1016/j.anai.2023.08.156.
